# Behavioral nudges and targeted education as sustainability strategies to reduce hazardous waste generation in intensive care and perioperative settings: a prospective interventional study

**DOI:** 10.1186/s44158-026-00368-w

**Published:** 2026-03-18

**Authors:** Giulia Roveri, Martina Vacondio, Ruth Martintoni, Kai Riemer, Matthias Bock, Simon Rauch

**Affiliations:** 1 Department of Anesthesia and Intensive Care Medicine, Hospital of Merano, Merano, Italy; 2 Institute of Mountain Emergency Medicine, Eurac Research, Bolzano, Italy; 3 Department of Psychology and Cognitive Science, University of Trento, Rovereto, TN Italy; 4 Fondazione Bruno Kessler, Povo, TN Italy; 5 Department of Anesthesia, Perioperative Medicine and Intensive Care, Paracelsus Medical University, Salzburg, Austria

**Keywords:** Hazardous waste, Healthcare waste management, Waste segregation, Sustainability, Behavioral nudges, Education

## Abstract

**Background:**

Intensive care units (ICUs) and operating rooms (ORs) are resource-intensive hospital areas and major contributors to healthcare waste. Proper segregation of hazardous and residual waste reduces carbon-intensive disposal and supports sustainability, yet practices depend heavily on staff behavior and knowledge.

**Methods:**

We conducted a prospective three-phase interventional study in the ICU and ORs of Merano Hospital, Italy (September 2023–May 2025). Baseline hazardous waste generation and staff knowledge/barriers were assessed (phase 1). Subsequently, low-cost behavioral nudges (enhanced bin visibility, labeling, placement–phase 2) and targeted online education on waste segregation (phase 3) were introduced in sequence. The primary outcome was the reduction in hazardous waste, normalized to ICU patient-days and surgical procedures; secondary outcomes included changes in perceived barriers and knowledge.

**Results:**

Hazardous waste generation declined across all phases in both ICUs and ORs. In the ICU, waste decreased from 3.31 (± 1.07) to 2.97 (± 1.40) kg/patient-day after behavioral nudges (− 10.3%, *p* = 0.31) and further to 1.97 (± 1.33) after education, representing a 40.6% reduction versus baseline (*p* < 0.001). In ORs, waste fell from 5.84 (± 1.40) to 4.38 (± 2.58) kg/procedure post-nudges (− 25.0%, *p* = 0.027) and to 3.84 (± 1.46) post-education, corresponding to a 34.4% reduction (*p* < 0.001). Structured questionnaires identified limited bin availability and unclear sorting rules as key barriers; behavioral nudges addressed structural obstacles, while education improved knowledge and confidence.

**Conclusions:**

Integrating low-cost behavioral nudges with targeted education effectively reduces hazardous waste in ICU and perioperative settings. Environmental changes improve waste practices, while education enhances staff knowledge, awareness, and confidence, emphasizing that infrastructure alone is insufficient without supportive training.

**Supplementary Information:**

The online version contains supplementary material available at 10.1186/s44158-026-00368-w.

## Introduction

The climate crisis assigns healthcare systems a dual responsibility: protecting human health and reducing their environmental impact [1]. Worldwide, healthcare is responsible for approximately 5% of greenhouse gas (GHG) emissions, with about 80% stemming from clinical care and just 20% from infrastructure such as buildings, electricity, and gas [2–4]. Intensive care units (ICUs) and operating rooms (ORs) contribute disproportionately to hospital waste due to their high patient acuity and procedural demands [3, 5]. ORs account for 20–33% of all hospital waste [6–9], and ICU patients produce 50% more solid waste and emit 200% more GHG emissions compared to patients on general wards [10].

Medical waste is generally divided into hazardous (infectious) and non-hazardous categories, and accurate sorting at the source is crucial, as each type requires specific disposal methods. The main challenge to efficient waste management is incorrect segregation [11, 12]. Although up to 90% of OR waste is nonhazardous and could potentially be recycled, 30–90% is incorrectly categorized and disposed of as hazardous, leading to notable environmental and financial consequences [8, 9]. Incinerating hazardous waste can generate up to ten times more emissions and costs 10–20 times more than disposing of nonhazardous waste [9, 13, 14], underscoring the critical need to reduce hazardous waste.

Although there are established rules and institutional protocols for the disposal of hospital waste, segregation practices frequently fail in practical, clinical environments [7]. Factors such as staff behavior, available resources, and awareness all influence compliance. However, little is known about healthcare workers’ actual knowledge of proper waste segregation or the barriers and psychological factors that influence their practices. Behavioral science suggests that environmental cues (nudges) and targeted training can promote sustainable practices, yet prospective interventional studies in ICU and perioperative settings remain scarce [15].

This study aimed to identify perceived barriers and knowledge gaps and to assess whether low-cost behavioral nudges combined with targeted education could reduce hazardous waste generation in ICU and OR settings.

## Methods

### Study design and setting

This prospective interventional study was conducted within the framework of the Green ICU initiative [5], which aims to implement evidence-based sustainability measures in high-intensity hospital environments. The study was conducted in the ICU and ORs at Merano Hospital, Italy, from September 2023 to May 2025, with no prior or concurrent institutional “green” initiatives or sustainability training in place. The ICU comprised a mixed medical-surgical ward with 8 intensive care beds and 6 intermediate care beds. The ORs handled a diverse spectrum of both planned and urgent surgeries covering most surgical disciplines, such as general surgery, gynecology and obstetrics, orthopedics, vascular surgery, urology, trauma, ENT, and ophthalmology, but did not include cardiac, neurosurgical, or thoracic procedures.

The Institutional Ethics Committee of Bolzano reviewed the study protocol and determined that formal ethics approval was not required, as the project was a quality improvement and service evaluation initiative without collection of health-related data. All staff involved in waste management in the ICU and ORs—including nurses, doctors, healthcare assistants, and cleaning personnel—were considered eligible to participate. This study was conducted and reported in accordance with the STROBE (Strengthening the Reporting of Observational Studies in Epidemiology) guidelines, and the corresponding checklist is provided as supplementary material.

### Study phases

**Fig. 1:**
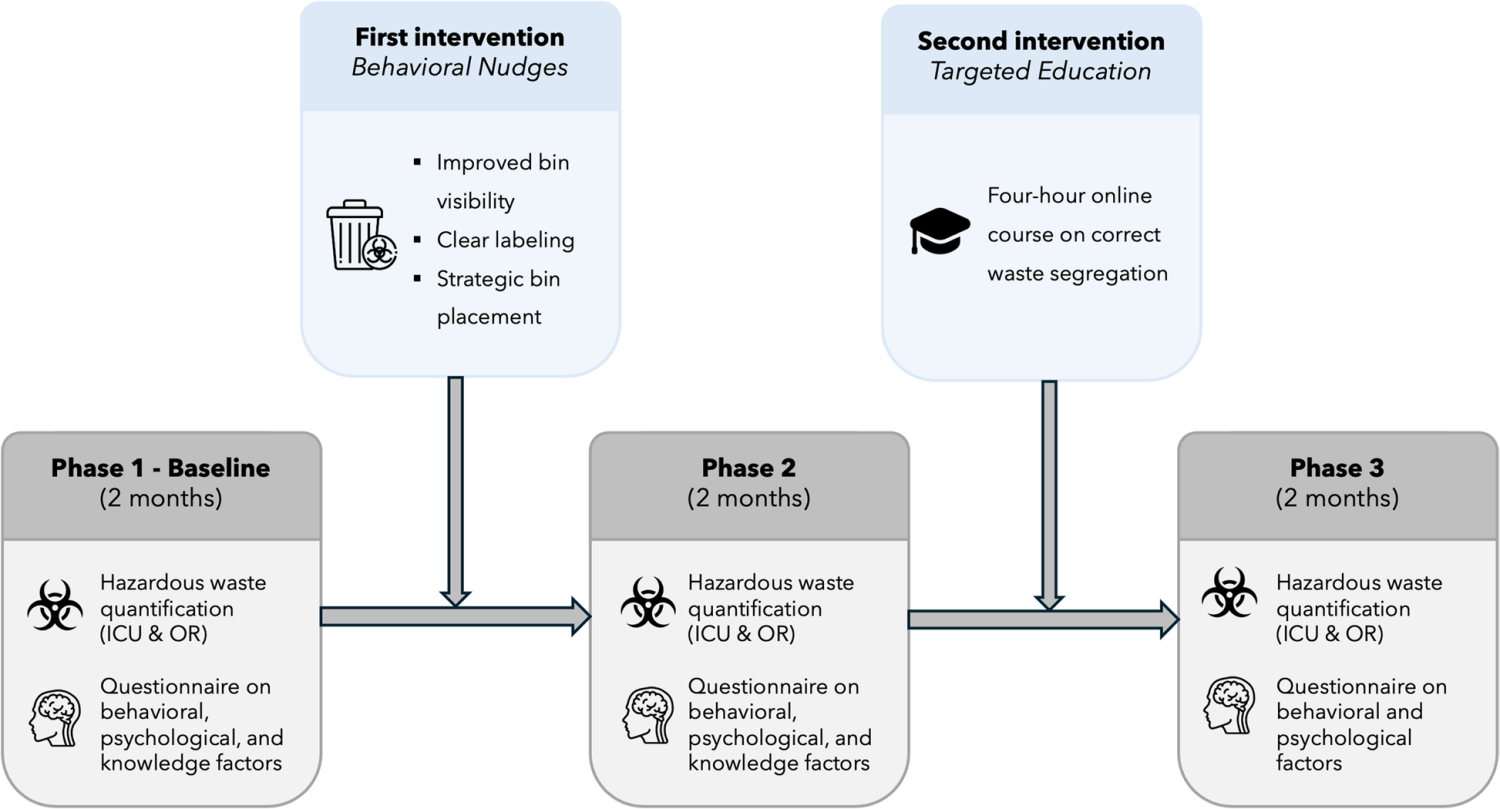
Timeline of the study. ICU: intensive care unit. OR: operating room

The project employed a three-phase pre–post intervention design (Fig. 1):

#### Phase 1—baseline

Over a 2-month period, hazardous waste generation was quantified separately for the ICU and ORs. Additionally, healthcare staff completed a survey evaluating behavioral and psychological factors as well as knowledge concerning waste segregation.

### *First intervention—behavioral nudge*

Low-cost behavioral nudges were implemented in ICU and ORs: (i) relocating waste bins to within immediate reach of care areas (bedside, preparation stations, and OR workspaces), (ii) increasing bin density to reduce walking distance to disposal points and (iii) applying standardized color-coding for each waste stream, (iv) adding large front-facing labels with text and pictograms. These modifications were introduced across the ICU and ORs to enhance visibility, accessibility, and ease of correct disposal.

#### Phase 2

Hazardous waste measurement was carried out again during a subsequent 2-month period, followed by a repeat administration of the behavioral, psychological, and knowledge questionnaire.

#### *Second intervention—targeted education*

Healthcare professionals participated in a 4-h, online, self-paced training course on appropriate waste management and segregation. The educational content was based on the World Health Organization (WHO) classification of healthcare waste and the Italian regulation DPR 254/2003, and covered waste categories, correct bin usage, common segregation errors, and local institutional procedures. The course was made available to all professional groups involved in ICU and operating room activities (physicians, nurses, healthcare assistants, and cleaning staff). Course completion was monitored, and only participants who completed the full training were included in the phase 3 analysis.

#### Phase 3

Hazardous waste generation was monitored again during another 2-month period, and the survey evaluating knowledge-related barriers (e.g., lack of awareness of the rules, absenceof instructions) factors was administered a third time.

### Outcomes

The main outcome measured was the variation in hazardous waste generated during the three different phases of the study. Additional outcomes involved changes in identified behavioral and psychological barriers and knowledge gaps that impact waste segregation.

### Waste quantification

Hazardous waste was defined based on the World Health Organization (WHO) classification for healthcare waste and the Italian regulation DPR 254/2003, and comprised infectious materials (such as items contaminated with blood) and sharps (including needles and scalpels) [16, 17]. Waste generation was recorded daily, separately for the ICU and ORs. For the ICU, waste generation (kg/day) was adjusted relative to the number of patients, while for the ORs, it was normalized by the number of surgeries performed. To facilitate consistent comparisons between phases of varying lengths, data were adjusted to a standardized 28-day month. Within this framework, weekdays were defined as days 1–5, 8–12, 15–19, and 22–26, and weekends as days 6–7, 13–14, 20–21, and 27–28. This standardization accounted for lower OR activity on weekends and maintained uniform operational patterns across all phases.

### Psychological, behavioral, and knowledge assessment

A survey distributed via the QualtricsXM platform was used to evaluate healthcare professionals’ psychological and behavioral attitudes regarding waste management. Participation was entirely voluntary, and informed consent was secured from all respondents. To track participants over time while maintaining their anonymity, individuals created unique identifiers based on non-identifiable personal details (such as certain letters from family members’ names and birthdates), enabling their responses to be linked across different study stages. The questionnaire, provided in both Italian and German, opened with clarifications of key operational terms. Specifically, “your workplace” referred to the ICU and/or OR at Merano Hospital, and “waste separation” followed the criteria outlined in DPR 254/2003.

To identify perceived barriers to appropriate waste segregation, participants completed two separate 7-item Likert scales (ranging from 1 = completely disagree to 7 = completely agree): one for hazardous waste and another for urban-like waste. The items addressed both logistical and cognitive obstacles, such as inadequate formal training, unclear rules, lack of space, time constraints, and concerns about safety.

Knowledge of correct waste segregation methods was assessed by 11 multiple-choice questions that addressed commonly used ICU and OR items, such as disposable linens, orthopedic casts, adult diapers, urine collection bags, glass vials for medication, IV fluids, bandages soiled with blood or pleural effusions, and cardboard boxes originating from infectious isolation rooms. Questions were developed following DPR 254/2003 and WHO recommendations [16, 17] and were administered consistently across all study phases.

### Statistical analysis

Pairwise Welch’s t-tests (assuming unequal variances) were used to examine changes in hazardous waste generation across the three study phases. A *p*-value of 0.05 or less was considered statistically significant.

We evaluated psychological and behavioral outcomes independently. To assess changes in perceived barriers and knowledge scores following the first and second interventions, linear mixed-effects models were applied. The model included phase as a fixed effect and participant ID as a random intercept to account for repeated measurements. Statistical significance was determined using Type III Wald chi-square tests.

All analyses were conducted using R version 4.1.3 (R Core Team, 2022). Results are presented as mean ± standard deviation (SD).

## Results

### Waste generation trends

ICU occupancy and surgical activity remained generally stable across the study phases. The mean number of ICU patients per day was 9.8 (± 1.4) in phase 1, 9.5 (± 1.4) in phase 2, and 7.2 (± 2.4) in phase 3. The mean number of surgical procedures per day was 21.2 (± 14.5) in phase 1, 20.4 (± 13.0) in phase 2, and 21.6 (± 16.4) in phase 3.

**Fig. 2:**
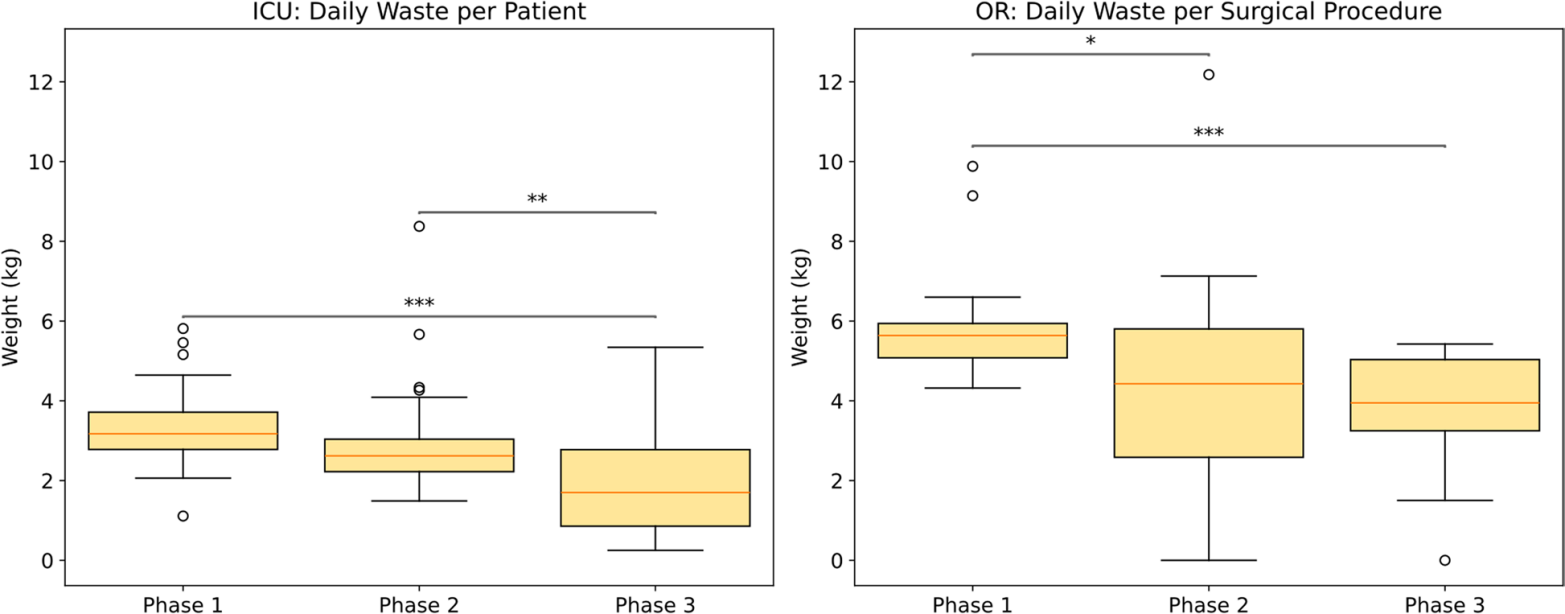
Amount of hazardous waste generated during the three study phases in the ICU (left panel; expressed as kilograms per day per patient) and in the ORs (right panel; expressed as kilograms per surgical procedure). *p < 0.05; **p < 0.01; ***p < 0.001

Hazardous waste generation declined across the three phases in both the ICU and the operating rooms (ORs) (Fig. 2). In the ICU, hazardous waste decreased from 3.31 ± 1.07 kg per patient per day in phase 1 to 2.97 ± 1.40 kg in phase 2, corresponding to a 10.3% reduction (95% CI − 30.9% to 10.2%, *p* = 0.31), and further declined to 1.97 ± 1.33 kg in phase 3, representing a 40.6% reduction relative to phase 1 (95% CI − 60.9% to − 20.4%, *p* < 0.001). In the ORs, mean hazardous waste decreased from 5.84 ± 1.40 kg per procedure in phase 1 to 4.38 ± 2.58 kg in phase 2, a 25.0% reduction (95% CI − 47.6% to − 2.4%, *p* = 0.027), and further declined to 3.84 ± 1.46 kg in phase 3, corresponding to a 34.4% reduction compared with phase 1 (95% CI − 50.5% to − 18.2%, *p* < 0.001).

### Psychological, behavioral and knowledge assessment

In total, 74 healthcare professionals from the ICU and OR at Merano Hospital took part in the first survey, with 26 completing the second and 8 completing the third survey. Demographic information and baseline characteristics are presented in Table 1.

**Table 1:** Demographic data and baseline characteristics of respondents from the first survey

Age (years), mean (SD)	44.3 (9.57)
Gender, N (%)	
Male	21 (28.4%)
Female	50 (67.6%)
Not reported	3 (4.1%)
Occupation, N (%)	
Physician	
*Anesthesiology and ICU*	*16 (21.6%)*
Nurse	
*Anesthesia Nurse*	*12 (16.2%)*
*Scrub Nurse*	*12 (16.2%)*
*ICU Nurse*	*18 (24.3%)*
Healthcare Assistant	10 (13.5%)
Cleaning Staff	6 (8.1%)
Primary working setting, N (%)	
Intensive Care Unit (ICU)	23 (31.1%)
Operating Room (OR)	27 (36.5%)
Both ICU and OR	24 (32.4%)
Years of work experience, mean (SD)	18.5 (9.73)

Results are expressed as mean (standard deviation SD) or number (percentage %)

*ICU* Intensive Care Unit, *OR* Operative Room

### Perceived barriers

At baseline (phase 1), participants indicated that they perceived generally low to moderate barriers to effectively separating waste, with some distinctions noted between urban-like waste and hazardous medical waste. For urban-like waste, the main obstacle identified was the distance between waste bins and the place where waste was produced (3.26 ± 2.10). This was followed by a lack of formal guidance on correct waste segregation (2.89 ± 2.23) and insufficient availability of bins (2.69 ± 2.13). The barrier reported least often was the belief that separating urban waste is unsafe (1.65 ± 1.27). Regarding hazardous medical waste, the primary obstacle reported was insufficient formal training (3.28 ± 2.38). Other difficulties mentioned were uncertainty about sorting rules (2.79 ± 1.98) and limited space for segregating waste (2.13 ± 1.75). As with urban waste, safety concerns were the least commonly mentioned barrier in handling hazardous medical waste (1.94 ± 1.54).

**Fig. 3:**
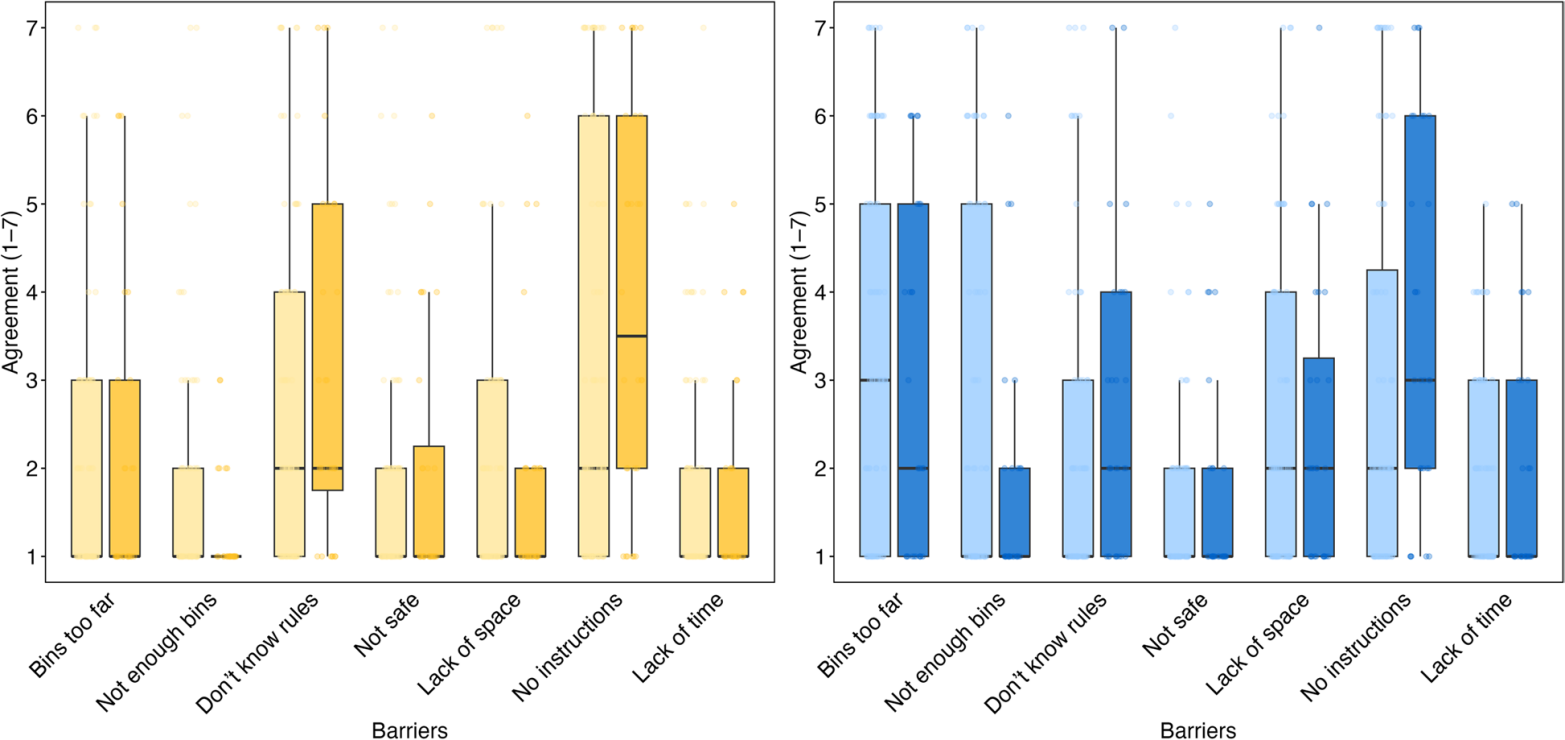
Perceived barrier to effective waste segregation during phase 1 and phase 2. The yellow chart on the left depicts hazardous waste, while the blue chart on the right illustrates urban-like waste. Phase 2 is indicated by a darker color, while phase 1 is shown with a lighter color. The graph displays data from the 26 participants who completed both phase 1 and phase 2

After implementing behavioral nudges (phase 2), two urban-like waste barriers showed statistically significant changes: participants indicated a significant decrease in viewing bin unavailability as a barrier (χ2(1) = 5.87, *p* = 0.015), and there was also a significant increase in recognizing lack of knowledge as an obstacle (χ2(1) = 4.59, *p* = 0.032). Figure 3 illustrates the perceived barrier to effective waste segregation in both phase 1 and phase 2.

**Fig. 4:**
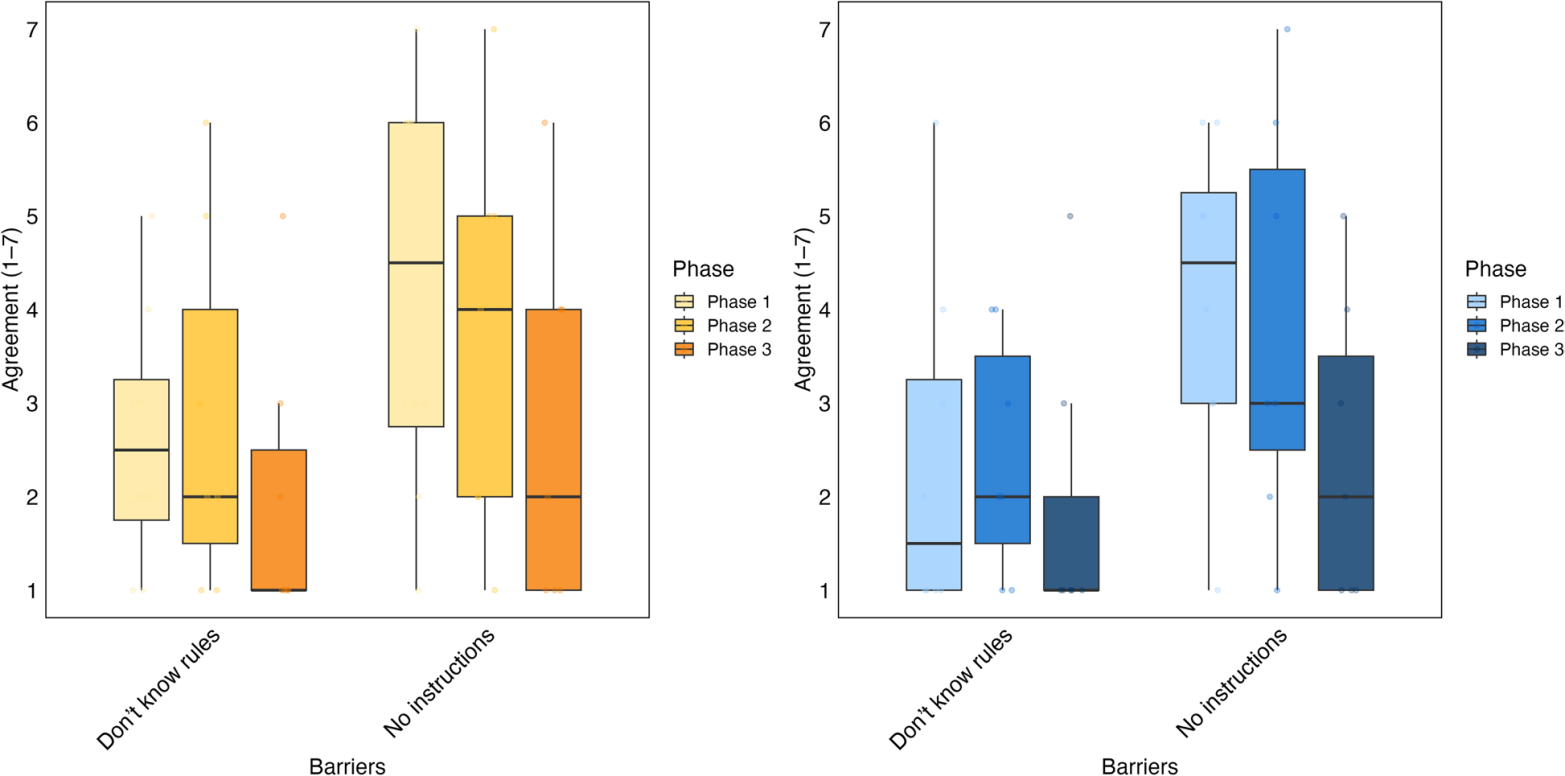
Perceived knowledge-related barriers to effective waste segregation addressed through targeted education. Barriers to proper waste segregation are highlighted for phase 1, phase 2, and phase 3. The yellow graph on the left presents hazardous waste data, while the blue graph on the right displays urban-like waste data. For each barrier, the boxplot on the far left (lightest color) corresponds to phase 1, the middle boxplot to phase 2, and the rightmost to phase 3. Results include data from the eight participants who completed all three phases

After the second, education-focused intervention, phase 3 showed a general reduction in the knowledge barriers targeted by the intervention (Fig. 4). For hazardous waste, participants reported that the perceived lack of formal instructions fell from 4.25 (± 2.25) in phase 1 to 2.71 (± 1.98) in phase 3. Additionally, their uncertainty regarding the rules dropped from 2.63 (± 1.41) to 2.00 (± 1.53). Urban-like waste revealed a comparable trend, with the lack of formal instructions decreasing from 4.13 (± 1.73) in phase 1 to 2.43 (± 1.62) in phase 3, and unclear rules reducing from 2.38 (± 1.85) to 1.86 (± 1.57).

### Knowledge

At baseline, participants exhibited a moderately high level of knowledge of proper waste segregation, achieving a mean score of 7.43 (± 1.56) out of a possible 11 points. After phase 2, knowledge scores showed minimal change (7.88 ± 1.36; compared to phase 1, χ2 (1) = 0.46, *p* = 0.499), while a slight increase was observed in phase 3 (8.67 ± 1.03).

## Discussion

This study’s results indicate that the implementation of behavioral nudges and targeted education reduced hazardous waste generation in both the ICU and OR. These findings align with earlier quasi-experimental and quality improvement research, which has demonstrated that modifying the environment and delivering repeated, customized education effectively enhances waste segregation practices [12, 18–20].

Consistent with previous studies [21–24], we found that both structural and knowledge-based challenges hindered effective reduction of hazardous waste in the ICU and OR environments. Initially, our study identified the primary obstacles such as the insufficient availability of appropriately labeled bins and the lack of formal training regarding correct hazardous material segregation. This is consistent with previous research indicating that misclassification is common. Up to 90% of waste from the OR is actually non-hazardous but is often managed as hazardous due to uncertainty about appropriate segregation guidelines [25].

After implementing low-cost behavioral nudges such as increasing bin visibility, using clear labels, and positioning bins strategically, we observed a decrease in perceived bin shortages. These straightforward environmental changes led to a reduction in hazardous waste production, which dropped by 10% in the ICU and 25% in the ORs between phase 1 and phase 2. Notably, following this intervention, participants reported greater awareness of gaps in their knowledge, suggesting that once structural obstacles were reduced, the complexity of proper waste sorting became more apparent. The absence of significant changes in overall knowledge scores between phases 1 and 2 also highlights the potential need for focused educational programs, such as those introduced in phase 3, to complement structural enhancements. After implementing a targeted educational intervention following phase 2, hazardous waste generation was further reduced, with reductions of 41% in the ICU and 34% in the OR when comparing phase 3 to phase 2. Furthermore, previously reported perceived barriers—for example, lack of knowledge of the rules and absence of formal instructions—also appeared to diminish.

In summary, our findings indicate that multifaceted interventions combining behavioral nudges with targeted education can contribute to a reduction in hazardous waste generation in the ICU and OR. Ongoing education, regular audits, and institutional commitment are required to maintain these gains and align hospital practices with broader sustainability goals [18, 20, 23, 24, 26].

### Limitations

Our study has several limitations. First, it was conducted at a single center, which may limit the generalizability of the findings to other hospital settings with different operational practices and cultural or regulatory environments. Second, the study did not employ a randomized controlled design or include a control group, so it is possible that unmeasured confounding factors could have contributed to the observed results. Third, reliance on self-reported data for behavioral outcomes and survey responses may introduce recall and social desirability bias, potentially overestimating true compliance and perceived improvement. Fourth, the follow-up period was relatively short, and the number of participants declined across successive survey phases, limiting the ability to assess long-term effects and the durability of the interventions. Fifth, the statistical analyses relied on mixed-effects models to account for repeated measures; however, these models are sensitive to missing data, unbalanced samples, and model assumptions, which may affect the robustness of estimated effects. Finally, the study focused on waste generation and perceived barriers but did not capture indirect environmental outcomes (e.g., carbon emissions), and granular data on costs and broader institutional sustainability metrics were not collected, limiting assessment of the economic and ecological impact of the interventions.

## Conclusion

In summary, integrating behavioral nudges with targeted education provides a low-cost, effective strategy to reduce hazardous waste in ICU and perioperative settings. Environmental changes improve practices, while education further strengthens staff knowledge, awareness, and confidence, underscoring that infrastructure alone is insufficient without complementary training.

## Supplementary Information

Supplementary Material 1.

## Data Availability

The datasets generated and analyzed during this study are available from the corresponding author upon reasonable request.

## Abbreviations

DPR: Decreto del Presidente della Repubblica (Italian regulation)
ENT: Ear, nose, and throat
GHG: Greenhouse gas
ICU: Intensive care unit
OR: Operating room
SD: Standard deviation
WHO: World Health Organization
