# Supplementary material for: Behavioral nudges and targeted education as sustainability strategies to reduce hazardous waste generation in intensive care and perioperative settings: a prospective interventional study
Source: J Anesth Analg Crit Care. 2026 Mar 18;6:47. doi: 10.1186/s44158-026-00368-w (PMC13011537; doi:10.1186/s44158-026-00368-w)
Supplement: Supplementary file 1 — Supplementary Material 1. [file 44158_2026_368_MOESM1_ESM.docx]

**STROBE Checklist**

Title of the study: *Behavioral Nudges and Targeted Education as Sustainability Strategies*

| Item No | STROBE Recommendation | Page/Section | Reported (Yes/No) |
| --- | --- | --- | --- |
| Title and abstract |  |  |  |
| 1a | Study design indicated in title | Title | Yes |
| 1b | Informative abstract with methods, results, conclusions | Abstract | Yes |
| Introduction |  |  |  |
| 2 | Background/rationale | Introduction | Yes |
| 3 | Specific objectives/hypotheses | Introduction | Yes |
| Methods |  |  |  |
| 4 | Study design presented early | Methods: Study Design and Setting | Yes |
| 5 | Setting, locations, dates | Methods: Study Design and Setting | Yes |
| 6 | Eligibility criteria, recruitment | Methods: Study Design and Setting | Yes |
| 7 | Outcomes, exposures, predictors | Methods: Outcomes | Yes |
| 8 | Data sources, measurement methods | Methods: Waste Quantification; Psychological, Behavioral and Knowledge Assessment | Yes |
| 9 | Bias addressed | Methods: Statistical Analysis | Yes |
| 10 | Study size rationale | Methods: Statistical Analysis | Yes |
| 11 | Quantitative variables handling | Methods: Statistical Analysis | Yes |
| 12 | Statistical methods, including secondary analyses | Methods: Statistical Analysis | Yes |
| Results |  |  |  |
| 13 | Participants at each stage | Results: Psychological, Behavioral and Knowledge Assessment | Yes |
| 14 | Descriptive data, participant characteristics | Results: Table 1 | Yes |
| 15 | Outcome data | Results: Waste Generation Trends | Yes |
| 16 | Main results, estimates with precision | Results: Waste Generation Trends, Figures 2–4 | Yes |
| 17 | Other analyses (subgroup, sensitivity) | Results: Psychological/behavioral analysis | Yes |
| Discussion |  |  |  |
| 18 | Key results summarized | Discussion | Yes |
| 19 | Limitations discussed | Discussion: Strengths and Limitations | Yes |
| 20 | Interpretation in context of other evidence | Discussion | Yes |
| 21 | Generalizability | Discussion: Strengths and Limitations | Yes |
| Other information |  |  |  |
| 22 | Funding sources and role | Funding section | Yes |
| 23 | Conflicts of interest | Conflicts of Interest section | Yes |

*to Reduce Hazardous Waste Generation in Intensive Care and Perioperative Settings: A Prospective Interventional Study*
